# Leaf extract of *Osbeckia octandra* induces apoptosis in oral squamous cell carcinoma cells

**DOI:** 10.1186/s12906-022-03505-4

**Published:** 2022-01-25

**Authors:** Jue Young Kim, Jin Kim, B. M. Ratnayake Bandara, Wanninayake M. Tilakaratne, Dokyeong Kim

**Affiliations:** 1 Oral Cancer Research Institute, Department of Oral Pathology, Yonsei University College of Dentistry, Seoul, 03722 Republic of Korea; 2 Department of Obstetrics and Gynecology, Gangnam Severance Hospital, Yonsei University College of Medicine, Seoul, 06230 Republic of Korea; 3 Department of Chemistry, Faculty of Science, University of Peradeniya, Peradeniya, 20400 Sri Lanka; 4 Department of Oral Pathology, Faculty of Dental Sciences, Center for Research in Oral Cancer, University of Peradeniya, Peradeniya, 20400 Sri Lanka; 5 Department of Oral Maxillofacial Clinical Sciences, Faculty of Dentistry, University of Malaya, Kuala Lumpur, 50603 Malaysia; 6 Precision Medicine Research Center, Department of Biomedicine & Health Sciences, College of Medicine, The Catholic University of Korea, Seoul, 06591 Republic of Korea

**Keywords:** *Osbeckia octandra*, Cell cycle arrest, Apoptosis, Oral squamous cell carcinoma

## Abstract

**Background:**

*Osbeckia octandra* is a plant endemic to Sri Lanka and is used in ethnomedicine for treating various diseases. However, the anti-cancer properties of *O. octandra* are yet to be fully investigated. In the present study, we evaluated the anti-cancer effects of *O. octandra* on oral cancer cells.

**Methods:**

Human oral cancer cell lines (HSC2, YD10B, YD38, YD9, and YD32) were used in this study. BrdU incorporation, cell cycle and annexin-V/PI staining were all evaluated using flow cytometry to determine the extent to which *O. octandra* leaf extract inhibits cell proliferation and induces apoptosis. Cell viability and reactive oxygen species (ROS) were also measured in order to investigate the anti-cancer effects of *O. octandra* extracts. Western blotting was performed to detect cell cycle related protein such as cyclin d1 and cdk4, and to detect apoptosis-related proteins such as Bcl-2, Bcl-X_L_, Bax, Caspase-9, Cleaved caspase-3, Fas, Caspase-8, and Bid.

**Results:**

Leaf extract of *O. octandra* reduced oral squamous cell carcinoma (OSCC) cell viability in a dose-dependent manner. Leaf extract of *O. octandra* has non-toxic in normal keratinocytes. Also, *O. octandra* extract interrupted the DNA replication via G1 phase arrests, and this effect was independent of ROS generation. In the apoptosis-related experiments, the population of annexin V-positive cells increased upon treatment with *O. octandra* extract*.* Furthermore, the expression of anti-apoptotic protein (Bcl-2 and Bcl-xL) was decreased, whereas the expression of cleaved caspase-3 protein was increased in *O. octandra-*treated OSCC cells.

**Conclusions:**

The results suggest that a leaf extract of *O. octandra* inhibited the proliferation of OSCC cells through G_1_ phase arrest and interrupting DNA replication. The leaf extract of *O. octandra* could trigger the apoptotic response via caspase 3 activation in OSCC cells. These results suggest that *O. octandra* has the potential to be developed as an alternative medicine for treating OSCC.

**Supplementary Information:**

The online version contains supplementary material available at 10.1186/s12906-022-03505-4.

## Background

Herbal extracts have long been used as a resource for developing therapeutics [1, 2] because of their long history of use and their typically low toxicity. In recent years, as people worldwide have been searching for more natural alternatives to treat diseases, herbal extracts have gained wide popularity as complementary and alternative medicines, especially in Asian countries [1, 3, 4].

The plant *Osbeckia octandra* (family Melastomataceae) is endemic to Sri Lanka, and is known as *Heen Bovitiya* locally [5]. The leaves and young stems are both edible, and the leaves, roots and bark are used in traditional medicines for treating diabetes mellitus, hepatitis, jaundice, hyperlipidemia, hemorrhoids and ascites. Additionally, the juice extracted from *O. octandra* leaves has protective effects against the liver damage induced by paracetamol poisoning [6], galactosamine and tert-butyl hydroperoxide [7]. The extracts of *O. octandra* have recently been shown to have anti-cancer effects on oral squamous cell carcinoma (OSCC), demonstrating that *O. octandra* can diminish OSCC cell migration and increase DNA fragmentation [8]. However, further studies are warranted to explore the effect of *O. octandra* on cancer cell proliferation and apoptosis. In this study, we found that the leaf extract of *O. octandra* hindered DNA replication via G_1_ phage arrest and caused apoptosis in OSCC cells.

## Methods

### Cell cultures

HSC2 OSCC cells were purchased from the Japanese Collection of Research Bioresources Cell Bank (Shinjuku, Japan), and the cells were maintained in F medium composed of Dulbecco’s modified Eagles medium (#12800–017, Gibco BRL, NY, USA) and F-12 Ham (#21700–075, Gibco BRL, NY, USA) mixed in a 3:1 ratio and supplemented with 10% fetal bovine serum and 1% penicillin/streptomycin. YD OSCC cell lines (YD9, YD32, YD38 and YD10B) were grown in F medium, supplemented with 1 × 10^− 10^ M cholera toxin (#C8052), 0.4 mg/ml hydrocortisone (#H4001), 5 μg/ml insulin (#I9278), 5 μg/ml transferrin (#T1147) and 2 × 10^− 11^ M triiodothyronine (T3, #T2877) [9]. The supplements to culture YD OSCC cell lines were purchased from Sigma Aldrich, Merck KGaA, Darmstadt, Germany. Human normal epidermal keratinocytes (HEK) were obtained from primary culture and used below the 9th passage. Ethically, the use of HEK cells was approved by the Institutional Review Board (IRB) of the Yonsei Dental Hospital, Yonsei University Health System, Seoul, Republic of Korea (IRB-2-2009-0002) as in our previous study [10]. HEK cells were grown in KGM medium (#CC-3107, Lonza, Basel, Switzerland). All the cells were kept in an incubator containing 5% CO_2_ at 37 °C; the culture medium was changed every 3 days.

### Preparation of *O. octandra* leaf extract

The leaves of *O. octandra* were collected using the correct collection permits by the curator of private garden in Kegalle, Sabaragamuwa Province, Sri Lanka. The botanical identity of the plant was confirmed by a field botanist (Mr. R. M. A. P. M. Rajatewa, Environmental Consultant, Educational and Rehabilitation Organisation, Gampaha, Sri Lanka) and authenticated at the Royal Botanical Gardens at Peradeniya, Sri Lanka; a voucher specimen (FAHS/OO/2013/01) of the plant has been deposited at the herbarium of the Department of Pharmacy, University of Peradeniya, Sri Lanka and is available for scientific studies upon request. The use of plant parts in the present study complies with international/national and institutional guidelines. The leaves were shade-dried for 6 weeks and ground to obtain a coarse powder using a mechanical grinder. The plant powder (50 g) was then shaken with dichloromethane and methanol (1:1, 1.5 l) on a bottle shaker at room temperature for 24 h. After decanting the solvent extract, the plant residue was extracted again with a fresh volume of solvent following the same procedure. The extracts were filtered using a Buchner funnel, filtrates combined, and the solvent evaporated to dryness in order to obtain the solvent-free dried extract. For in vitro experiments, the dried extract was dissolved in DMSO (#D1370, Duchefa Biochemie, RV Haarlem, Netherlands) and the supernatant was used in the assays described below.

### Cell viability and cytotoxicity assay

Cells were seeded into 96-well culture plates (2 × 10^3^/well) and treated with the indicated concentrations (50 to 300 μg/ml) of *O. octandra* leaf extract for 72 h before MTT (#M1415, Duchefa Biochemie, RV Haarlem, Netherlands) assays were performed to measure cell viability.

### BrdU incorporation

After treatment with the *O. octandra* extract, a BrdU incorporation assay was performed to determine the frequency at which cells were synthesizing DNA. BD Pharmingen™ BrdU flow kits (#559619, BD Biosciences, San Jose, CA, USA) were used following the manufacturer’s instructions. In brief, cells were plated at 1 × 10^6^ cells/100 mm culture dish. Each plate was then maintained in the presence or absence of *O. octandra* leaf extract (100 μg/ml) for 24 h before the BrdU solution was carefully added directly to each culture medium. After incubation, BrdU stained cells were measured by flow cytometry (Becton Dickinson, Beckman Coulter) as per manufacturer’s instruction. The DNA synthetic activity of cells was determined by analyzing the correlated expression of total DNA (7-AAD staining) and incorporated BrdU levels.

### Cell cycle analysis using flow cytometry

Cell cycle analysis was carried out using propidium iodide (PI) staining. The cells were plated to 1 × 10^6^ cells/100-mm culture dish and stabilized overnight. The next day, the cells were treated with *O. octandra* extract (100 μg/ml) for 12 h or 72 h before being harvested and fixed with ethanol in PBS. After centrifuging the cells, the cell pellets were incubated with an RNase A (#10109169001, Roche, Basel, Switzerland) solution at 37 °C for 30 min to which PI staining solution (50 μg/ml) was added. The cell cycle distribution was then analyzed by flow cytometry (BD FACSCanto II flow cytometer, BD Biosciences, Franklin Lakes, NJ, USA).

### Reactive oxygen species (ROS) generation

The generation of ROS was measured using flow cytometry [10]. Briefly, cells (4 × 10^5^ cells/6-well plate) were grown with or without *O. octandra* extract (100 μg/ml) for 24 h and then 10 μM 2′,7′-dichlorofluorescin diacetate (H_2_DCFDA) (#D399, Molecular Probes Inc., Eugene, OR, USA) dissolved in DMSO was applied. After incubating at 37 °C for 20 min, the cells were detached and analyzed by flow cytometry (Beckman Coulter, Fullerton, CA, USA).

### Protein extraction and western blotting

Cells (1 × 10^6^ cells/100-mm culture dish) were seeded and allowed to stabilize overnight. Cells were then lysed by using cell lysis buffer (#9803, Cell Signaling Technology, Danvers, MA, USA) containing phenylmethanesulfonyl fluoride (PMSF, #78830, Sigma Aldrich, Merck KGaA, Darmstadt, Germany) and the lysates were put on ice for 30 min. After vortex mixing, the cell lysates were swirled every 5 min. Lastly, the lysates were centrifuged, and the supernatant was stored at − 20 °C until use. To perform the western blot, the obtained supernatants were mixed with 5X sodium dodecyl sulfate (SDS) sample buffer and loaded onto an SDS-PAGE gel. After running the gel, the protein bands were transferred to a PVDF membrane and blocked with 5% skim milk in PBS with Tween 20 added. After blocking, the membranes were incubated with indicated primary antibodies at the appropriate diluted ratio given below.

The antibodies against Cyclin D1 (#2978 s, 1:1000, Cell Signaling Technology, Danvers, MA, USA), CDK4 (#ab108357, 1:1000, Abcam, Cambridge, UK), Bcl-2 (#sc-7382, 1:500, Santa Cruz Biotechnology, Dallas, TX, USA), Bcl-xL (#sc-8392, 1:500, Santa Cruz Biotechnology, Dallas, TX, USA), Bax (#ab32503, 1:1000, Abcam, Cambridge, UK), Caspase-9 (#9502 s, 1:1000, Cell Signaling Technology, Danvers, MA, USA), and Cleaved Caspase-3 (Asp175, 1:1000, Cell Signaling Technology, Danvers, MA, USA), Fas (#8023 s, 1:1000, Cell Signaling Technology, Danvers, MA, USA), Caspase-8 (#9746 s, 1:1000, Cell Signaling Technology, Danvers, MA, USA), and Bid (#2002p, 1:1000, Cell Signaling Technology, Danvers, MA, USA) were used. The secondary antibodies (anti-rabbit- (#7074S) and anti-mouse-IgG, HRP-linked (#7076S)) were purchased from Cell Signaling Technology (Danvers, MA, USA). β-Actin (#BS6007M, 1:5000) that was purchased from Bioworld Technology, Inc. (St. Louis, MN, USA) was used as a housekeeping control.

### Annexin V and PI staining using flow cytometry

YD10B and HSC2 cells (5 × 10^5^) were grown in 100-mm dishes incubated at 37 °C overnight. The cells were then treated with the *O. octandra* extract for the indicated period. Afterwards, the cells were stained using an annexin V-fluorescein isothiocyanate (FITC) kit (#556547, BD Biosciences, San Jose, CA, USA) according to the manufacturer’s protocol. Briefly, both types of cells were washed by cold PBS and then the cells were incubated with FITC annexin V and/or PI in 1X Binding Buffer for at room temperature 15 min. The stained cells were then analysed by flow cytometry. For bar graphs, the fraction of early apoptotic cells was measured by detecting cells stained only with annexin V. A FC500 series CXP cytometer and CXP analysis (Beckman Coulter, USA) were used for measurement and data analysis, respectively.

### Statistical analysis

All statistical analyses were performed using SPSS version 20 (SPSS Inc., Chicago, IL, USA). Differences between groups were analyzed using Mann-Whitney U tests. Each experiment was performed at least in triplicate. The results are reported as the mean ± standard deviation (SD). A value of *P*-value < 0.05 was considered statistically significant.

## Results

### Leaf extract of *O. octandra* reduced the viability in OSCC cells

**Fig. 1:**
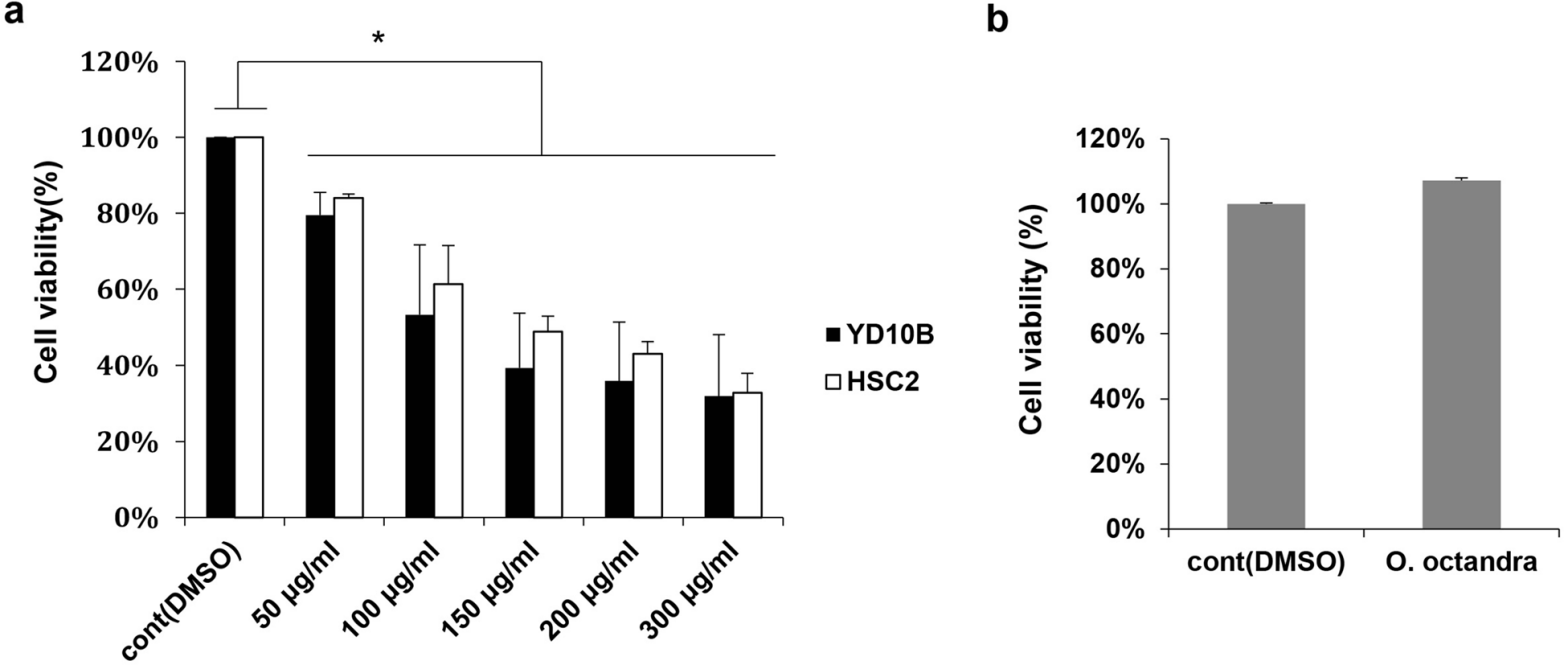
Cell viability with *O. octandra* treatment in OSCC cells and HEK cells. **a** OSCC cells were seeded in 96-well culture plates (2 × 10^3^/well) and were treated with the indicated concentrations (50 to 300 μg/ml) of *O. octandra* extract for 72 h before MTT assay was performed. The extract reduced the cell viability of HSC2 and YD10B cells in a dose-dependent manner. **b** HEK cells were seeded into 96-well culture plates (2 × 10^3^/well) and treated with 100 μg/ml of *O.octandra* extract for 72 h before MTT assay was performed (* *P* < 0.05, Mann whitney U test)

To investigate the cytotoxicity of *O. octandra* extract to OSCC cells, we measured cell viability after treatment with 50–300 μg/ml *O. octandra* extract for 72 h, using MTT assay. Compared with the control (DMSO), the treatment of *O. octandra* extract significantly reduced the viability of various OSCC cell lines (* *P* < 0.05) (Fig. 1a and Additional file 1: Fig. S1). Among them, the viabilities of YD10B and HSC2 cells were markedly reduced by the *O. octandra* extract in a dose-dependent manner (Fig. 1a). A concentration of 100 μg/ml *O. octandra* extract, which induced about 50% cell death in both types of OSCC cells, was selected for subsequent assays. There was no noticeable difference in cell viability between untreated- and extract*-*treated human-normal keratinocytes (HEK) cells (Fig. 1b). Taken together, *O. octandra* extract displayed cytotoxicity only to OSCC cells and was not toxic to normal keratinocytes.

### Leaf extract of *O. octandra* inhibited the proliferation of OSCC cells via cell cycle arrest in the G1 phase

**Fig. 2:**
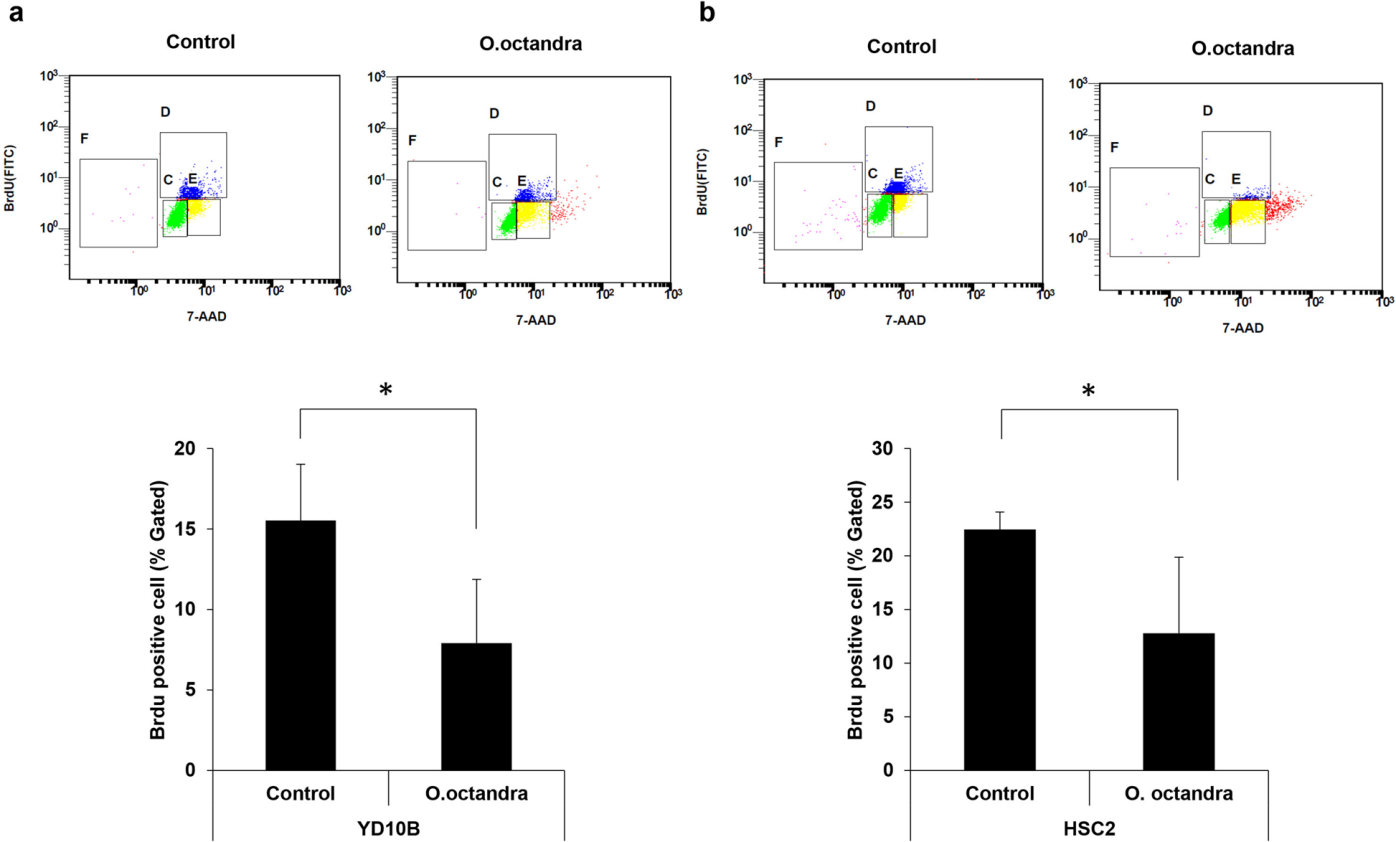
The leaf extract of *O. octandra* interrupts DNA replication in OSCC cell. **a** and **b** YD10B (**a**) and HSC2 (**b**) cells were plated at 1 × 10^6^ cells/100-mm culture dish. Each well was maintained in the presence or absence of *O. octandra* (100 μg/ml) for 24 h before carefully adding the BrdU solution directly into the cultured medium. After incubation, BrdU staining was performed. The DNA synthetic activities of cells can be determined by analyzing the correlated expression of total DNA (7-AAD staining) and incorporated BrdU levels. The BrdU-positive cells in S-phase are represented by blue spots (upper panels) and their quantity is indicated by bar graphs (lower panels) (* *P* < 0.05, Mann whitney U test)

**Fig. 3:**
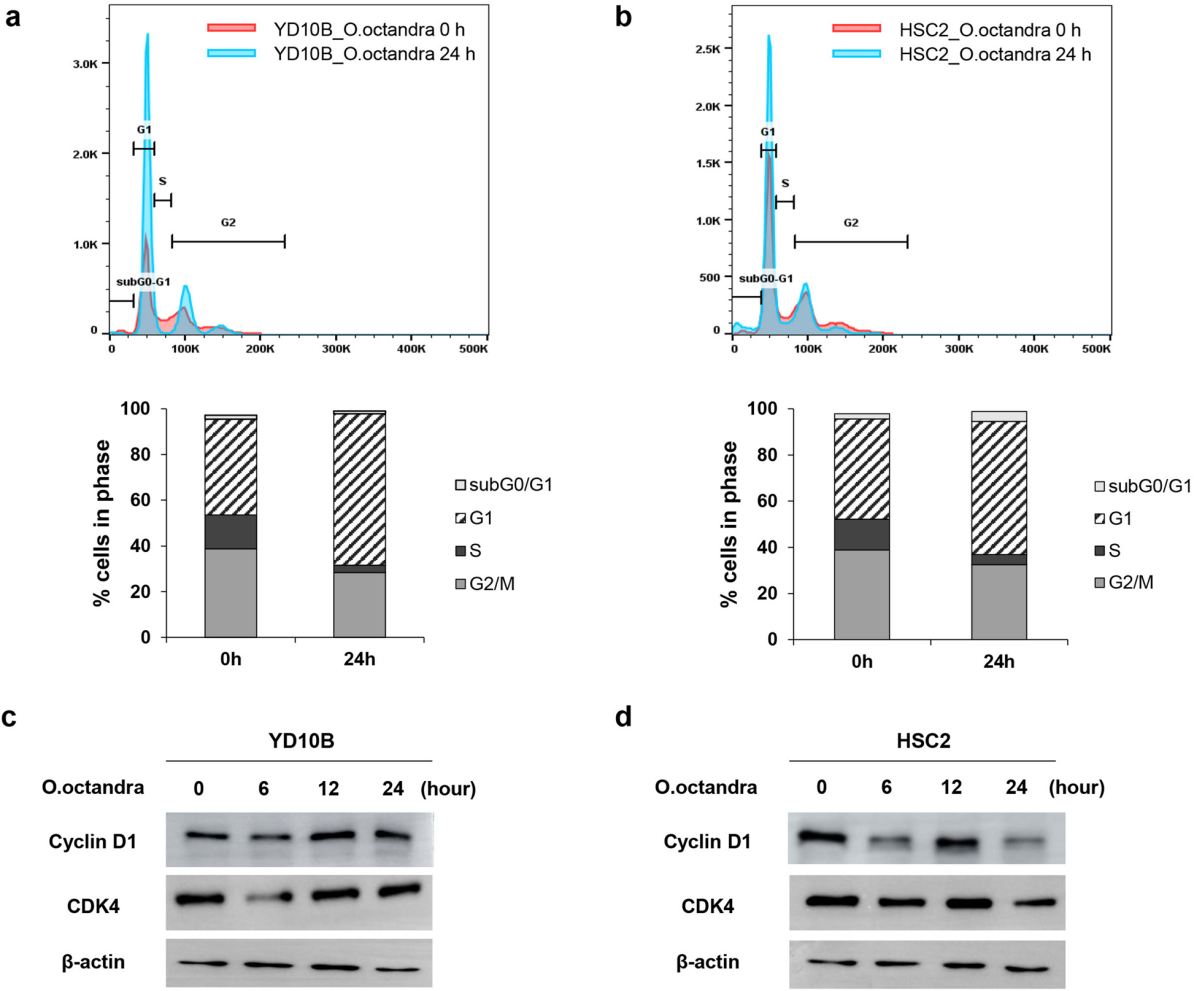
The leaf extract of *O. octandra* induces cell cycle arrest in OSCC cell. **a** and **b** YD10B (**a**) and HSC2 (**b**) cells were plated at 1 × 10^6^ cells/100-mm culture dish, and then treated with 100 μg/ml *O. octandra* extract for the indicated time. Propidium iodide staining was performed, and the stained cells were analysed by flow cytometry to evaluate the cell cycle. The histograms for cell cycle analysis are represented at 24 h after treatment with *O. octandra* extract (upper panels; red line: 0 h skyblue line: 24 h) and their quantity is indicated by bar graphs within cell cycle phase (lower panels) (*** *P* < 0.001, Mann whitney U test). The details were shown in Table 1. **c** and **d** YD10B (**c**) and HSC2 (**d**) cells were seeded at 1 × 10^6^ cells/100-mm culture dish. After stabilizing for 24 h, the cells were treated with 100 μg/ml *O. octandra* extract for the indicated time and harvested. Proteins were then extracted from the harvested cell lysates (* *P* < 0.05). Protein bands were separated on the same gel and were cropped. Full length of immunoblots was shown in Additional file 5: Fig. S5

To investigate whether *O. octandra* extract would interrupt the DNA replication of OSCC cells, BrdU staining was performed. Our results clearly show that *O. octandra* extract interrupted the DNA replication of OSCC cells (Fig. 2a and b), decreased the DNA replication of YD10B cells from 15.53 ± 3.50% (DMSO) to 7.91 ± 3.96% (extract) (* *P* < 0.05), and decreased the replication of HSC2 cells from 22.45 ± 1.63% (DMSO) to 12.79 ± 7.07% (extract) (* *P* < 0.05). We further verified the growth inhibitory activity of *O. octandra* extract on OSCC cells via cell cycle analysis. After treatment with *O. octandra* extract for 24 h, our results show that extract-stimulated YD10B cells increased the proportion of cells in the G1 phase to 66.17 ± 2.37% (*** *P* < 0.001) (Fig. 3a and Table 1). Similarly, extract-stimulated HSC2 cells increased the proportion of cells in the G_1_ phase to 57.63 ± 0.40% (Fig. 3b and Table 1), compared with control cells (*** *P* < 0.001). Furthermore, both cell types showed an increase in sub G_0_/G_1_ phase cells and a decrease in S phase cells. The tendency is consistent with an increase in G_1_ phase cells and a decrease in S phase cells at 6 h after treatment with *O. octandra* extract (Table 1). It is indicating that *O. octandra* leaf extract increased the population of apoptotic cells. Additionally, we investigated the effect of *O. octandra* extract on the expression of cell cycle regulatory genes, which play a central role in the cell cycle progression and induced G_1_ phase arrest. *O. octandra* extract resulted in a clear down-regulation in the protein expression levels of cyclin D1 at 6 h after treatment with *O. octandra* in both types of OSCC cells (Fig. 3c and Additional file 5: Fig. S5). Similarly, a marked decreased in the expression of CDK4 was detected at 6 h in both types of OSCC cells. These results showed that *O. octandra* extract decreased the protein expression of cyclin D1-CDK4 complex, confirming that *O. octandra* extract induces cellular apoptosis via cell cycle arrest in the G1 phase.

**Table 1:** Cell cycle profiles of OSCC cells treated with *O. octandra* extracts

**Cell cycle profiles of YD10B cells treated with** ***O. octandra***			
	**Time**		
	**T0 h**	**T6 h**	**24 h**
Sub G0/G1	1.77 ± 0.25	3.63 ± 4.55	1.21 ± 0.20
G1	41.83 ± 4.58	52.03 ± 13.55	66.17 ± 2.37
S	14.87 ± 2.23	15.77 ± 2.40	3.25 ± 1.95
G2/M	38.77 ± 0.76	27.83 ± 15.93	28.40 ± 4.44
**Cell cycle profiles of HSC2 cells treated with** ***O. octandra***			
	**Time**		
	**0 h**	**6 h**	**24 h**
Sub G0/G1	2.30 ± 0.17	6.41 ± 1.83	4.26 ± 2.76
G1	43.37 ± 1.44	47.90 ± 5.74	57.63 ± 0.40
S	13.40 ± 0.60	9.86 ± 0.09	4.40 ± 0.47
G2/M	38.87 ± 1.23	34.63 ± 7.70	32.53 ± 2.75

Each value represents mean ± SD of each performed in triplicated. *SD* Standard deviation

To understand how *O. octandra* leaf extract could trigger G_1_ phase arrest and lead to apoptosis, we examined ROS generation; the latter could cause DNA damage and cell cycle arrest in a variety of conditions. Unexpectedly, the treatment with *O. octandra* extract decreased ROS generation in YD10B cells (4.63-fold decrease) and HSC2 cells (1.79-fold decrease), respectively (* *P* < 0.05) (Additional file 2: Fig. S2), suggesting that the extract of *O. octandra* functions as an antioxidant and does not cause ROS-induced DNA damage.

In summary, these results suggest that *O. octandra* extract mediates G_1_ phase arrest, thereby retarding cell proliferation and subsequently promoting cellular apoptosis. These processes were not caused by *O. octandra*-stimulated ROS generation; on the contrary, the leaf extracts of *O. octandra* extract are known to have antioxidant properties in vitro [11].

### Leaf extract of *O. octandra* caused apoptosis via caspase-3 cascade in OSCC cells

**Fig. 4:**
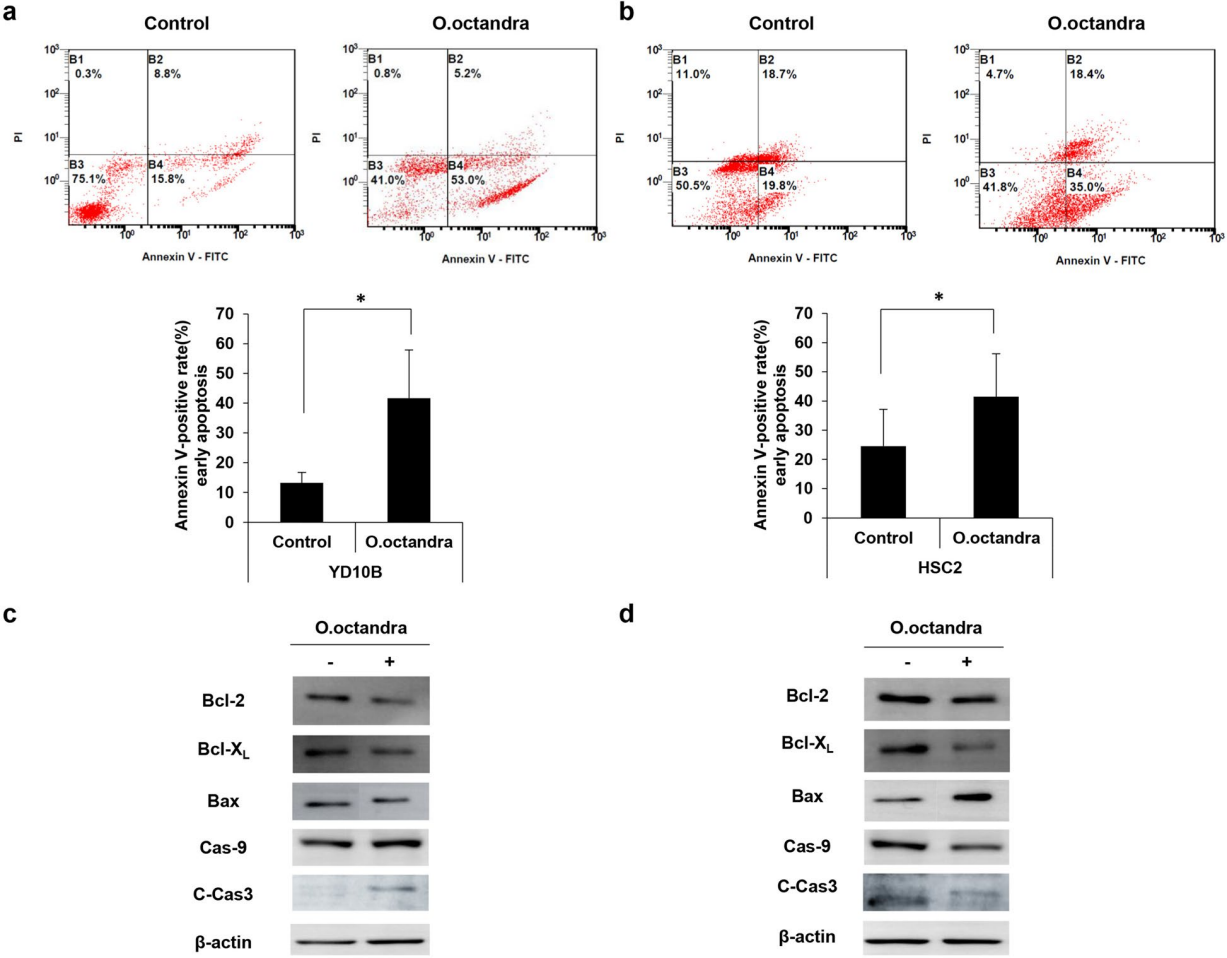
The apoptotic effects of *O. octandra* leaf extract in OSCC cell. **a** and **b** YD10B (**a**) and HSC2 (**b**) cells (5 × 10^5^) were grown in 100-mm dishes and incubated at 37 °C overnight. The cells were then treated with 100 μg/ml *O. octandra* extract for 24 h. Afterwards, the cells were stained with an annexin V-fluorescein isothiocyanate. The annexin V-positive cells (early apoptotic cells) are represented within B4 area in scatter graph (upper panels) and their quantity is indicated by bar graphs (lower panels) (* *P* < 0.05). **c** and **d** YD10B (**c**) and HSC2 (**d**) cells were seeded at 1 × 10^6^ cells/100-mm culture dish. After stabilizing for 24 h, the cells were treated with 100 μg/ml *O. octandra* extract for the indicated time and harvested. Proteins were then extracted from the harvested cell lysates (* *P* < 0.05). Protein bands were separated on the same gel and were cropped. Cropped blots for Bcl-2, Bcl-X_L_, Cas-9 (Caspase-9), and C-Cas-3 (Cleaved Caspase3) are shown at 6 h after treatment with 100 μg/ml *O. octandra* and cropped blots for Bax are shown at 24 h after treatment with 100 μg/ml *O. octandra* in each OSCC cells. Uncropped blots were shown in Additional file 6: Fig. S6

To determine if *O. octandra* extract could induce apoptosis, we first performed annexin V and PI staining and the stained cells were analysed by flow cytometry. The proportion of annexin V-positive YD10B cells increased to 41.67 ± 16.21% (extract-treated) compared with control (DMSO-treated) cells (13.17 ± 3.57%) (* *P* < 0.05) (Fig. 4a). Additionally, the proportion of annexin V-positive HSC2 cells increased to 41.45 ± 14.74% compared with control (DMSO-treated) cells (24.53 ± 12.63%) (* *P* < 0.05) (Fig. 4b). These results suggest that *O. octandra*-induced cell death is caused by apoptosis in OSCC cells.

Further, we investigated the effect of the leaf extract on the apoptosis-regulating protein, including Bcl-2, Bcl-X_L_, Bax, Caspase-9, and Cleaved caspase-3. The expression of anti-apoptotic Bcl-2 and Bcl-X_L_ was decreased 6 h after treatment with *O. octandra* extract in both types of OSCC cells, whereas *O. octandra* extract treatment was clearly increased the expression of pro-apoptotic Bax after treatment with them in HSC2 cells. Lastly, *O. octandra* extract increased cleaved caspase-3 in both types of OSCC cells (YD10B and HSC2), compared with control cells (Fig. 4c, d and Additional file 6: Fig. S6). Additionally, we investigated whether *O. octandra* extract can be initiated through activation of the extrinsic pathway (Additional file 3: Fig. S3 and Additional file 4: Fig. S4). As results, the protein levels of Fas were weakly increased at 24 h after treatment with *O. octandra* extract in only HSC2 OSCC cells, not in YD10B cells. Moreover, there is no tendency in protein expression of caspase-8 and bid, which are the downstream of extrinsic pathway mediated by the death receptor. Taken together, these results suggest that cell death induced by *O. octandra* leaf extract is caused by intrinsic apoptotic pathway through caspase-3 activation and the Bax/Bcl-X_L_ and Bcl-2 ratio in OSCC cells.

## Discussion

OSCC is the most common malignant tumor among head and neck cancers and has a high rate of morbidity and mortality. For treatment in patient with OSCC, several therapies are used, which include conventional therapies such as surgery, chemotherapy, radiotherapy, or a combination of these depending on the tumor type. Unfortunately, these therapies come with abundant complications and only a moderate success rate [12]. For instance, chemotherapeutic drugs can exacerbate the hematologic toxicities like anemia, leukopenia and thrombocytopenia [13]. Additionally, the combinations of chemotherapy, radiotherapy and surgery could produce adverse effects on orofacial regions, such as hyposalivation, fibrosis of the temporomandibular joint (TMJ), chemotoxicity in the jaw, and fungal and/or bacterial infections of the mucosa [14]. Thus, alternative medicines with low toxicity would bring in numerous benefits in cancer therapy.

The plant *O. octandra* is used in traditional herbal medicine for treating jaundice and other liver diseases [5–7]. Recently, a leaf extract of *O. octandra* has shown in vitro anti-cancer effects on OSCC cells by reduction of cell migration and induction of DNA damage [8]. Whether *O. octandra* can induce apoptosis in OSCC cells remains to be explored. To this end, we performed various in vitro experiments to demonstrate the apoptotic effects of *O. octandra* leaf extract on OSCC cells. Our data reveal that *O. octandra* decreased OSCC cell viability and DNA replication while no significant damage was observed in HEK cells. Moreover, the treatment with *O. octandra* extract increased G_1_ cell populations. Thus, the cell death was most likely caused by G_1_ arrest in both types of OSCC cells. Interestingly, G_1_ checkpoint arrest might also result in dissipating an exogenous cellular stress signal [15]. In addition to the G_1_ checkpoint arrest, the *O. octandra* extract also increased the sub G_0_/G_1_ cell population transiently*,* suggesting that the extract induced cellular apoptosis. Consistent with these observations, G_1_ checkpoint signaling could result in the activation of pathways such as the p53 pathway, also leading to programmed cell death [15]. Similarly, our finding shows that S checkpoint was upregulated in concomitant with G_1_ checkpoint arrest. Cyclin D1, with its partner CDK4/6, plays a dominant role in regulating G_1_/S cell cycle progression of various cancer cells [16, 17]. Consistent with cell cycle profiles, we demonstrated that downregulation of cyclin D1 and CDK4 at an earlier (6 h) treatment time point after treatment with *O. octandra* extracts, meaning that *O. octandra* extracts is sufficient to induce G_1_ cell cycle arrest in OSCC cells.

G_1_ arrest is involved in the apoptotic pathways [18]. Conversely, we have previously reported that DNA damage is induced by ROS generation in OSCC carcinogenesis [10]. Based on the previous contention, we investigated whether *O. octandra*-induced DNA damage [8] is dependent with ROS. Contrary to expectations, *O. octandra* extracts suppressed the generation of ROS, indicating that *O. octandra* functions as an antioxidant [11]. Taken together, these data suggest that the *O. octandra-*mediated DNA damage and G_1_ checkpoint arrest were independent of ROS suppression. Our data show that the *O. octandra* extract functions as an antioxidant to diminish ROS generation in OSCC cells. Next, we observed that the treatment with *O. octandra* extract increased annexin V-positive cells and induced apoptosis via induction of caspase-3 in both types of OSCC cells. Consistent with these observations are data that suggest that high amounts of antioxidants can lead to cellular apoptosis via caspase cascade in OSCC cells [19, 20]. Apoptosis of tumor cell is a complex process involving the regulation of numerous genes, including members of the Bcl-2 family, which are among the most important members as they are able to inhibit the apoptosis of cells [21]. The mitochondrial apoptosis pathway is mediated by anti-apoptotic and pro-apoptotic proteins, including the Bcl-2 family and Bax proteins [21, 22]. Thus, we observed the protein expression of Bcl-2 family proteins, including Bcl-2, Bcl-XL, and Bax. The protein levels of Anti-apoptotic Bcl-2 and Bcl-X_L_ were shown at an earlier (6 h) treatment time point after treatment with *O. octandra* extracts in both types of OSCC cells. Conversely, the protein expression of pro-apototic Bax was only shown increased levels at 12, 24 h after treatment with *O. octandra* extracts in HSC2 cells. There is no alteration of Bax protein in YD10B cells. However, the ratio of pro-apoptotic protein/anti-apoptotic protein could facilitate the cellular apoptosis [23, 24], thus *O. octandra* extract-induced cell death is involved in cellular apoptosis. We further investigated whether *O. octandra* extract-induced apoptosis is involved in extrinsic or intrinsic apoptosis pathway. Apoptosis can be initiated through activation of the extrinsic (receptor) pathway or intrinsic pathway [21]. In extrinsic pathway, activation of death receptors, such as tumor necrosis factor (TNF) – α and Fas with its ligand FasL, triggers the formation of a death-inducing signaling complex (DISC) involving the recruitment of the adaptor protein FADD. Following its recruitment, it activates caspase-8, in turn can cleave apoptotic substrates like caspase-3 and bid [25, 26]. Our findings provide that a weakly increased expression of Fas in HSC2 cells, whereas a decreased expression of Fas in YD10B cells, compare with control cells (0 h). Additionally, we cannot observe the tendency by *O. octandra* extract in protein expression of caspase-8 and bid, meaning that *O. octandra* extract-induced apoptosis is not involved in extrinsic pathway. Furthermore, an increase of caspase-9 protein was shown at 12 h in YD10B cells and at 24 h in HSC cells, respectively, but cleavage of caspase-9 was not detected. Although caspase-9 is an upstream effector of caspase-3 in intrinsic apoptosis pathway, the protein expression of cleaved caspase-3 was increased at an earlier time point (from 6 h after treatment with *O. octandra* extract) than at time point when increased expression of caspase-9 was shown. It is indicated that caspase-3 can be activated by *O. octandra* extract-triggered several signals such as caspase-2/7, BNIP, Bcl-2 family protein, NF-κB and MAPK [21, 27–29]. Taken together, *O. octandra* extract-induced apoptosis is not involved in death receptor mediated extrinsic pathway. However, it is involved in caspase-3 cascade and it might be regulated by internal stimuli such as DNA damage and by Fas/Caspase-8 independent pathway [27]. To clear the hypothesis, further studies are required for in-depth understanding of the signaling pathway of apoptosis induced by the *O. octandra* extract.

Collectively, *O. octandra* extract increased cellular apoptosis through G_1_ cell cycle arrest and a caspase 3 cascade-dependent pathway in OSCC cells. However, our research has some study limitations. We only used one type of normal cells, and thus other types of normal cells should also be examined to unequivocally establish that *O. octandra* leaf extract is not toxic to normal cells. Furthermore, we did not identify what components from *O. octandra* extract can induce apoptosis. The leaf of *O. octandra* contains phenols, tannins, flavonoids, alkaloids and terpenoids [8]. Flavonoids function as antioxidants [30] and can induce G_0_/G_1_ arrest and apoptosis through p38/JNK MAPKinase in hepatocellular carcinoma cells [31]. Thus, MAPKinase might be involved in cellular apoptosis induced by the *O. octandra* extract in OSCC cells. Additionally, terpenoids can also induce cell cycle arrest and apoptosis, and increase the amount of ROS as well [32]. In addition, phenols, tannins and alkaloids have also been implicated in anticancer and antioxidant activity of phytochemicals [33–35]. However, the specific bioactive compounds of *O. octandra* that induce cell cycle arrest and apoptosis in OSCC cells are still unknown. Thus, further studies are required to identify the specific compounds that confer anticancer and antioxidant properties to the leaf extract of *O. octandra.*

## Conclusion

A leaf extract of *O. octandra* inhibited the proliferation of OSCC cells in vitro through cell cycle arrest in G_1_ phase and interrupting DNA replication; the extract (≤ 100 μg/ml) had little or no cytotoxicity in normal keratinocytes. The leaf extract of *O. octandra* could trigger the apoptotic response via caspase 3 activation in OSCC cells. These findings could lead to the development of novel herbal medicines from *O. octandra*, specifically targeting OSCC cells.

## Supplementary Information

**Additional file 1.**

**Additional file 2.**

**Additional file 3.**

**Additional file 4.**

**Additional file 5.**

**Additional file 6.**

**Additional file 7.**

## Data Availability

The datasets generated and analyzed during the current study are available from the corresponding author on reasonable request.

## Abbreviation

OSCC: Oral squamous cell carcinoma
