# Supplementary material for: Leaf extract of Osbeckia octandra induces apoptosis in oral squamous cell carcinoma cells
Source: BMC Complement Med Ther. 2022 Jan 25;22:20. doi: 10.1186/s12906-022-03505-4 (PMC8787916; doi:10.1186/s12906-022-03505-4)
Supplement: Supplementary file 1 — Additional file 1. [file 12906_2022_3505_MOESM1_ESM.pdf]

Supplementary figures and figure legends

Additional file 1: Fig. S1

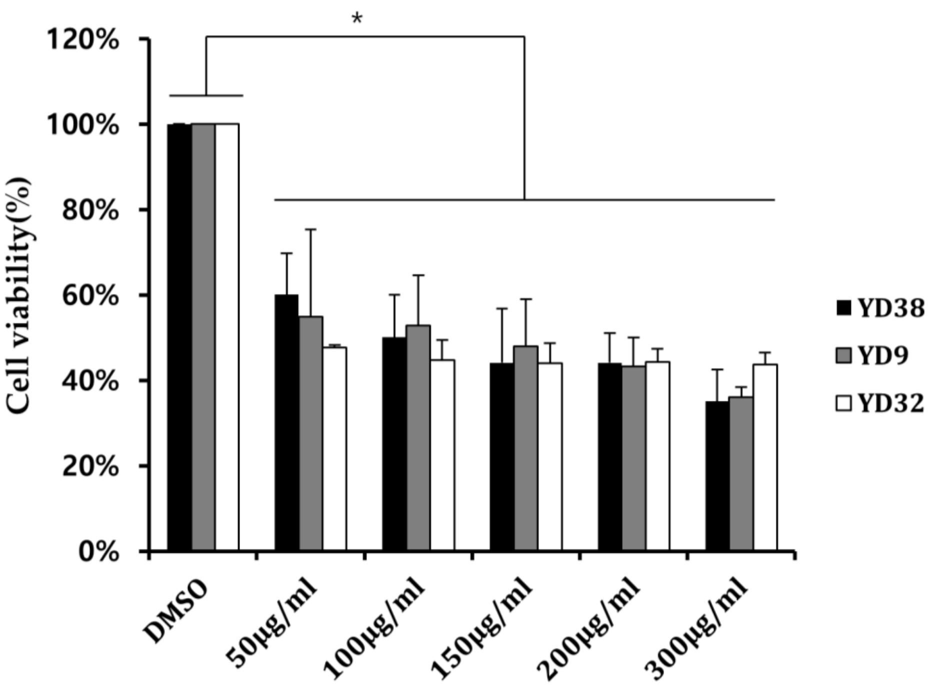

Fig. S1. OSCC cell viability by treatment with *O. octandra*. Three OSCC cells (YD38, YD9 and YD32) seeded in 96-well culture plats (2 x 10^3/well) and were treated with the indicated concentractions (50 µg to 300 µg/ml) of *O. octandra* for 72 h, and the MTT assays were performed (*P < 0.05 by Mann-Whitney U tests).
