# Supplementary material for: Leaf extract of Osbeckia octandra induces apoptosis in oral squamous cell carcinoma cells
Source: BMC Complement Med Ther. 2022 Jan 25;22:20. doi: 10.1186/s12906-022-03505-4 (PMC8787916; doi:10.1186/s12906-022-03505-4)
Supplement: Supplementary file 2 — Additional file 2. [file 12906_2022_3505_MOESM2_ESM.pdf]

Supplementary figures and figure legends

Additional file 2: Fig. S2

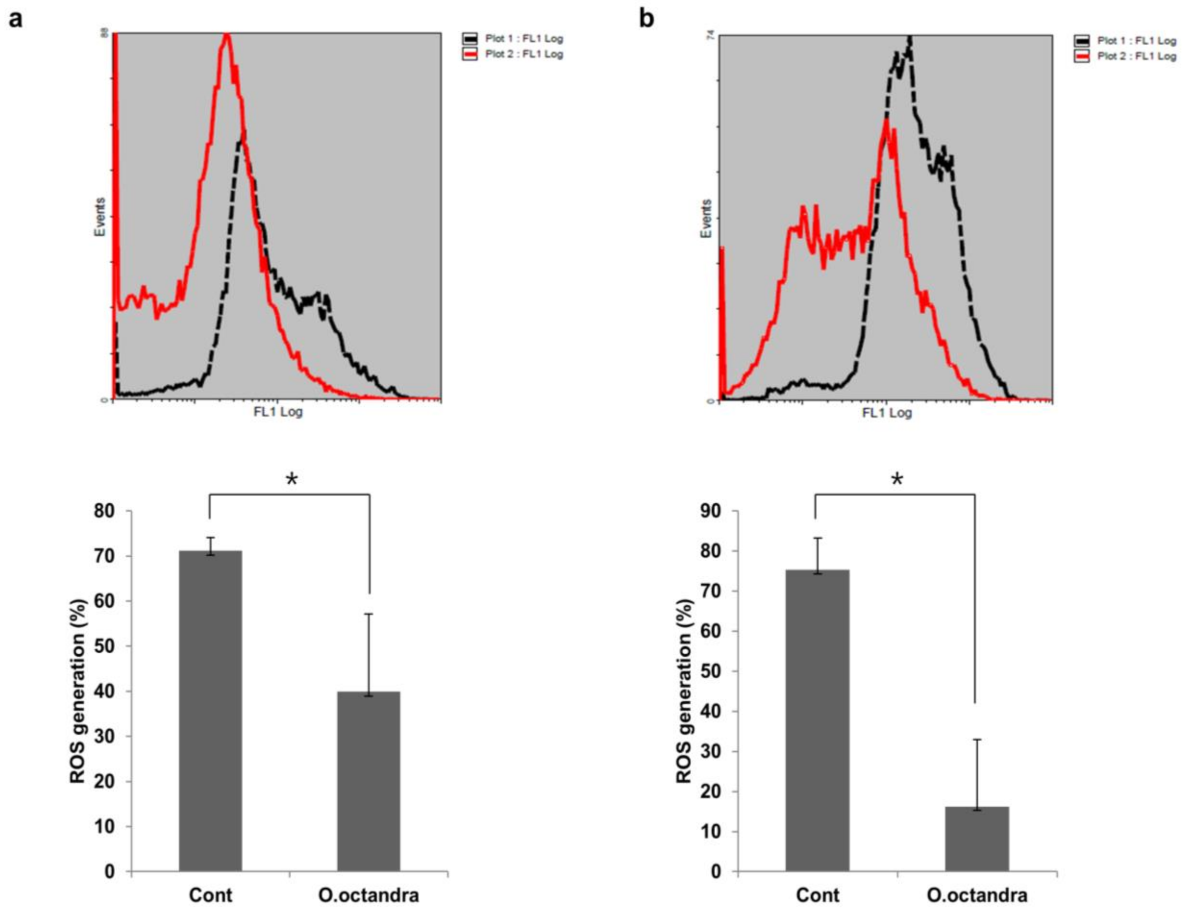

Fig. S2. The extracts of *O. octandra* reduce ROS generation in OSCC cells. **a** and **b**. YD10B (**a**) cells and (**b**) HSC cells were seeded into 6 well plates with or without *O. octandra* (100μg/ml) for 24 h, thereby both cells applied with the 10 μM of fluorescent probe 2'7'-dichlorofluorescin diacetate (H_2DCFDA) dye was applied in the cells at 37 °C for 20 min. The representative histograms indicate black line (control; untreated OSCC cells) and Red line (*O. octandra* treated OSCC cells), respectively. The bar graphs were analyzed by Mann-Whitney *U* tests (**P* < 0.05).
