# Supplementary material for: Leaf extract of Osbeckia octandra induces apoptosis in oral squamous cell carcinoma cells
Source: BMC Complement Med Ther. 2022 Jan 25;22:20. doi: 10.1186/s12906-022-03505-4 (PMC8787916; doi:10.1186/s12906-022-03505-4)
Supplement: Supplementary file 3 — Additional file 3. [file 12906_2022_3505_MOESM3_ESM.pdf]

Supplementary figures and figure legends

Additional file 3: Fig. S3

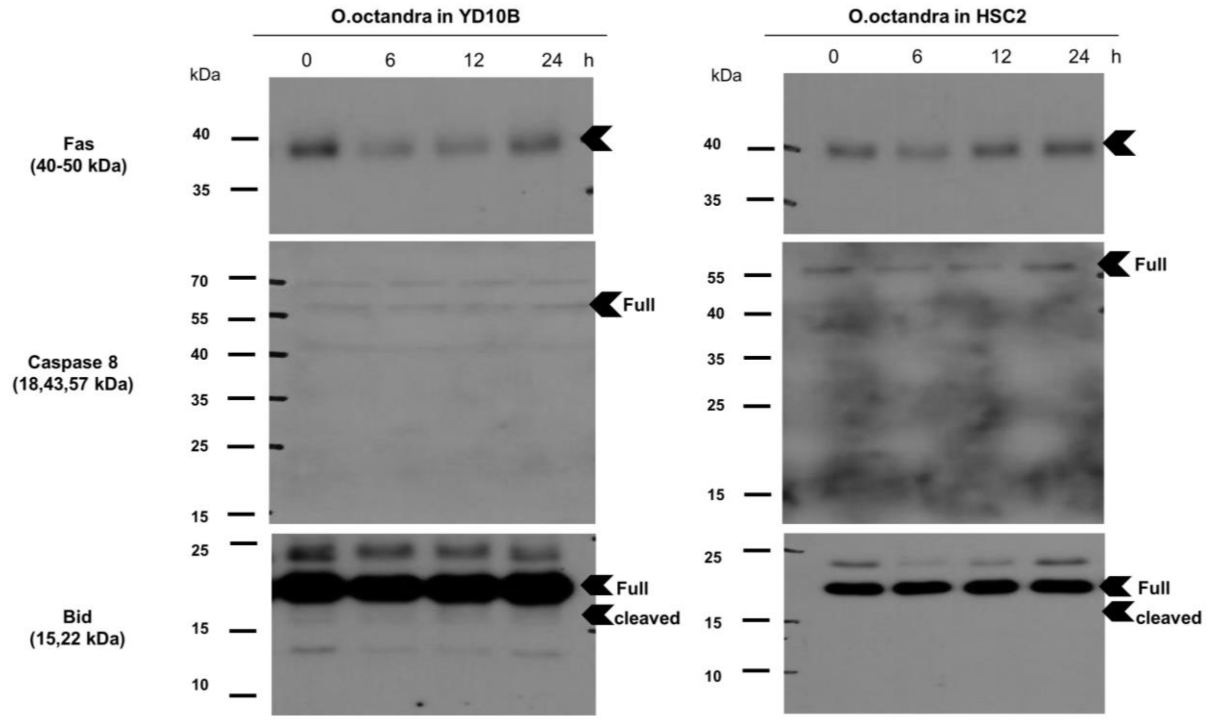

Fig. S3. Extrinsic apoptosis-related protein expression of Fas, Caspase-8, and Bid. Both OSCC cells were treated with *O. octandra* for indicated time (6, 12, 24 h). Protein samples were run in three identical sets and transferred to PVDF membranes. Membranes were probed with the indicated primary and secondary antibodies. Primary antibodies against Fas (#8023s, 1:1000), Caspase 8 (#9746s, 1:1000), and Bid (#2002p, 1:1000) were obtained from Cell Signaling Technology (Danvers, MA, USA).
