# Supplementary material for: Leaf extract of Osbeckia octandra induces apoptosis in oral squamous cell carcinoma cells
Source: BMC Complement Med Ther. 2022 Jan 25;22:20. doi: 10.1186/s12906-022-03505-4 (PMC8787916; doi:10.1186/s12906-022-03505-4)
Supplement: Supplementary file 4 — Additional file 4. [file 12906_2022_3505_MOESM4_ESM.pdf]

Supplementary figures and figure legends

Additional file 4: Fig. S4

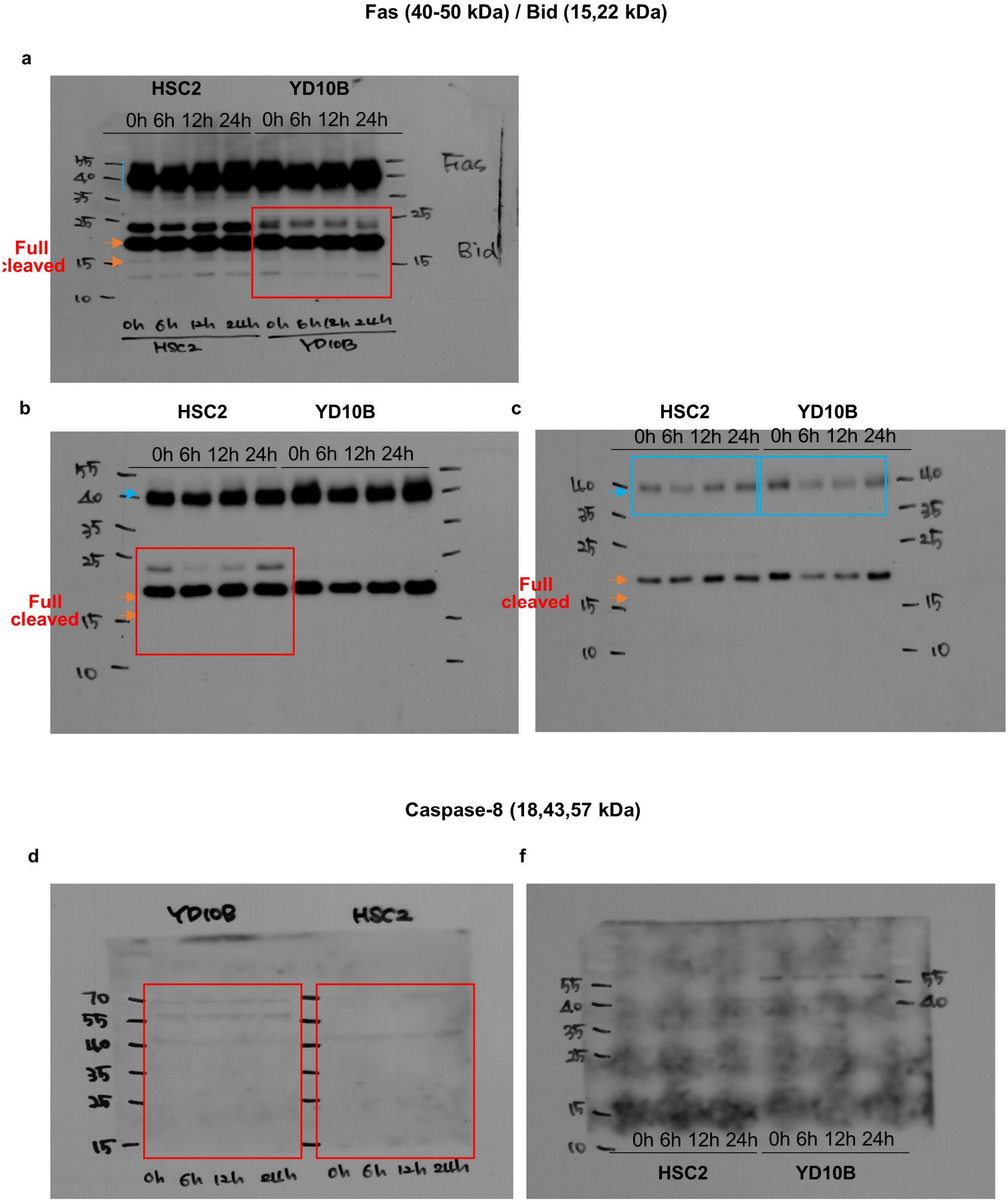

Fig. S4. Full length immunoblots of Fas, Caspase-8, and Bid in **Additional file 3: Fig. S3.**
