# Supplementary material for: Leaf extract of Osbeckia octandra induces apoptosis in oral squamous cell carcinoma cells
Source: BMC Complement Med Ther. 2022 Jan 25;22:20. doi: 10.1186/s12906-022-03505-4 (PMC8787916; doi:10.1186/s12906-022-03505-4)
Supplement: Supplementary file 5 — Additional file 5. [file 12906_2022_3505_MOESM5_ESM.pdf]

Supplementary figures and figure legends

Additional file 5: Fig. S5

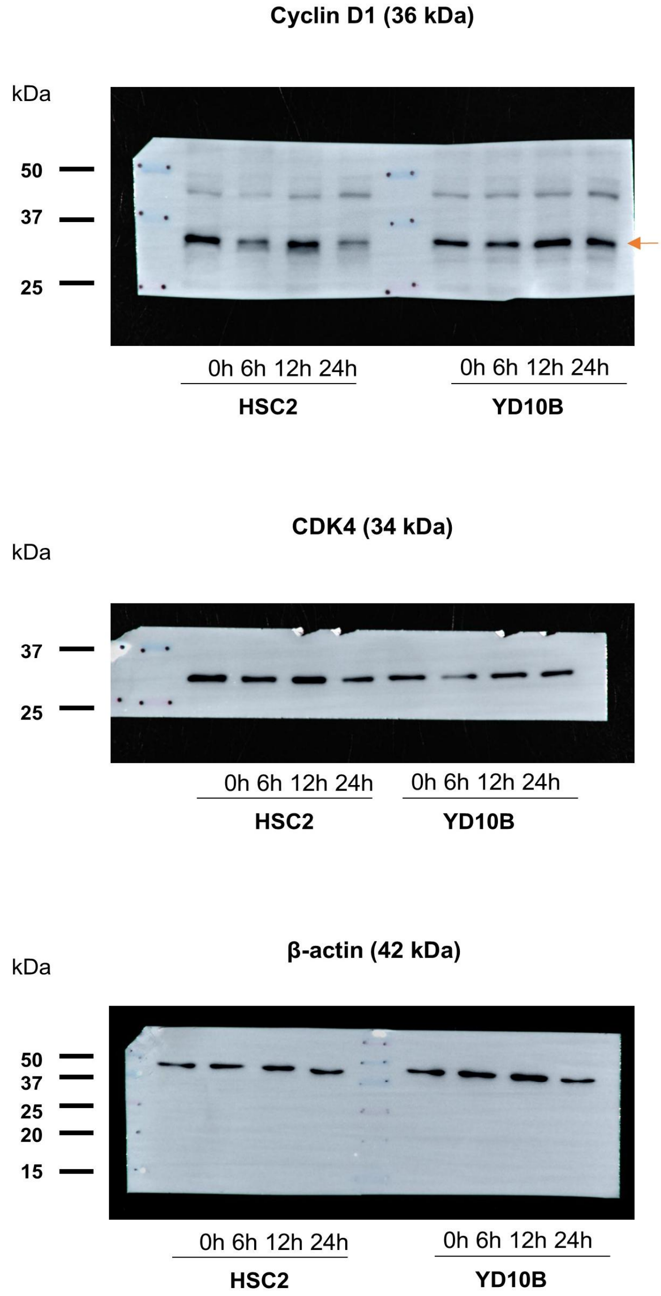

Fig. S3. Full length immunoblots of Cyclin D1, Cdk4, and β-actin in **Fig. 3c** and **Fig. 3d.** Both OSCC cells were treated with *O. octandra* for indicated time (6, 12, 24 h). Protein samples were run in three identical sets and transferred to PVDF membranes. Membranes were probed with the indicated primary and secondary antibodies.
