# Supplementary material for: Leaf extract of Osbeckia octandra induces apoptosis in oral squamous cell carcinoma cells
Source: BMC Complement Med Ther. 2022 Jan 25;22:20. doi: 10.1186/s12906-022-03505-4 (PMC8787916; doi:10.1186/s12906-022-03505-4)
Supplement: Supplementary file 6 — Additional file 6. [file 12906_2022_3505_MOESM6_ESM.pdf]

Supplementary figures and figure legends

Additional file 6: Fig. S6

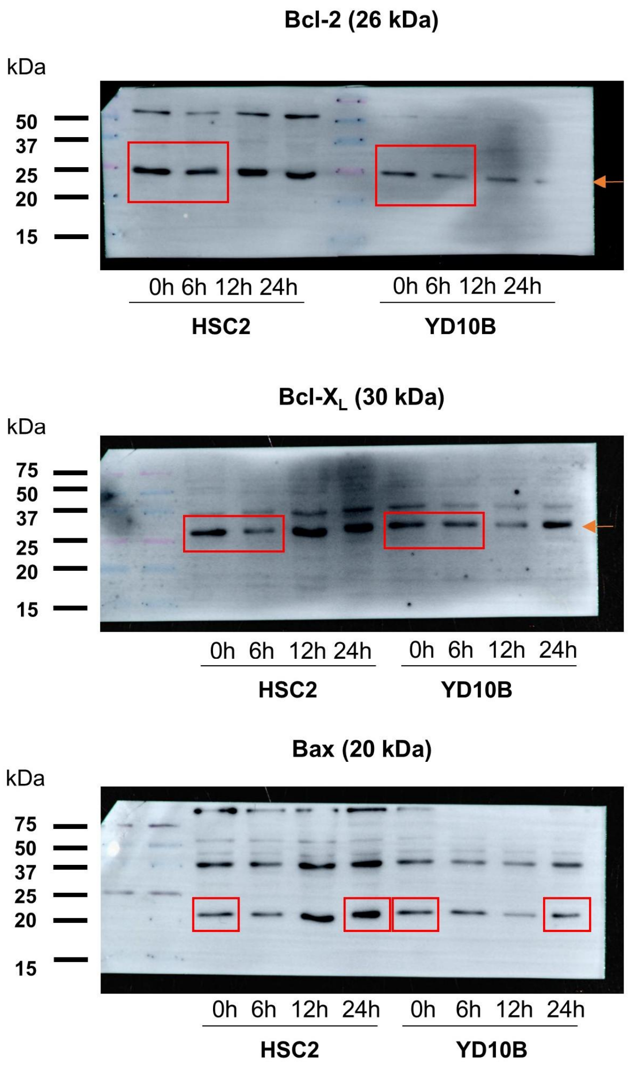

Caspase-9 (Cas-9)
(35,37,47 kDa)

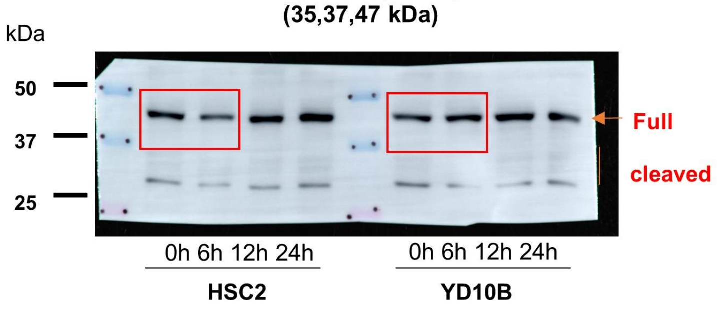

Cleaved Caspase3 (C-Cas3)
(17,19 kDa)

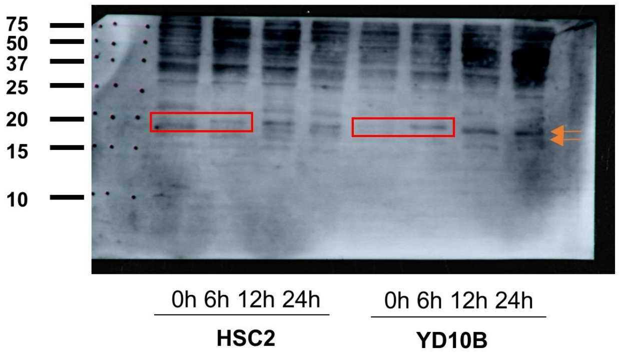

β-actin (42 kDa)

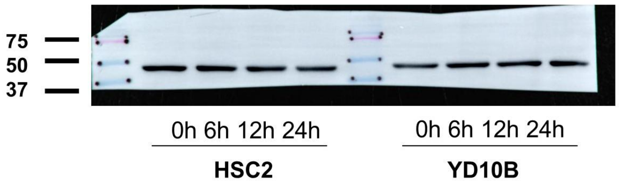

Fig. S4. Full length immunoblots of Bcl-2, Bcl-X_L, Bax, Caspase-9 (Cas-9), Cleaved Caspase-3(C-Cas3) and β-actin in **Fig. 4c** and **Fig. 4d.** Both OSCC cells were treated with *O. octandra* for indicated time (6, 12, 24 h). Protein samples were run in three identical sets and transferred to PVDF membranes. Membranes were probed with indicated primary and secondary antibodies. The red rectangle represents the cropping area.
