# Supplementary material for: Leaf extract of Osbeckia octandra induces apoptosis in oral squamous cell carcinoma cells
Source: BMC Complement Med Ther. 2022 Jan 25;22:20. doi: 10.1186/s12906-022-03505-4 (PMC8787916; doi:10.1186/s12906-022-03505-4)
Supplement: Supplementary file 7 — Additional file 7. [file 12906_2022_3505_MOESM7_ESM.docx]

***Supplementary data for***

**Leaf extract of *Osbeckia octandra* induces apoptosis in oral squamous cell carcinoma cells**

Jue Young Kim^1,2^, Jin Kim^1^, B. M. Ratnayake Bandara^3^, Wanninayake M. Tilakaratne^4,5^ and Dokyeong Kim^1,6*^

^1^Oral Cancer Research Institute, Department of Oral Pathology, Yonsei University College of Dentistry, Seoul 03722, Republic of Korea

^2^Department of Obstetrics and Gynecology, Gangnam Severance Hospital, Yonsei University College of Medicine, Seoul 06230, Republic of Korea

^3^Department of Chemistry, Faculty of Science, University of Peradeniya, 20400 Peradeniya, Sri Lanka

^4^Department of Oral Pathology, Faculty of Dental Sciences, Center for Research in Oral Cancer, University of Peradeniya, 20400 Peradeniya, Sri Lanka

^5^Department of Oral Maxillofacial Clinical Sciences, Faculty of Dentistry, University of Malaya, Malaysia

^6^Precision Medicine Research Center, Department of Biomedicine & Health Sciences, College of Medicine, The Catholic University of Korea, Seoul 06591, Republic of Korea

**Correspondence**

Dokyeong Kim, Ph.D., ^6^Precision Medicine Research Center, Department of Biomedicine & Health Sciences, College of Medicine, The Catholic University of Korea, Seoul 06591, Republic of Korea. Phone:82-2-2258-7352; E-mail: dkkim2908@gmail.com

**Supplementary Materials and Methods**

**Cell culture**

YD9, YD32, and YD38 cells were maintained in EF medium (the mixture of F medium and E medium at 9: 1 ratio). The details for growth medium were described in original materials and methods. All cells were grown at 37℃ in a humidified incubator with 5% CO_2_. Three OSCC cells lines were obtained from department of oral pathology, Yonsei University College of Dentistry.

**Cell cytotoxicity test**

For cytotoxicity test, cells (2 x 10^3^) were seeded into the individual wells of a 96-well plate and treated with *O. octandra* leaf extract (50 to 300 μg/ml) in serum-free medium. After 72 h, MTT (#M1415, Duchefa Biochemie, RV Haarlem, Netherlands) assays were conducted. The optical density was measured at 570 nm using a microplate reader. All experiments were performed in triplicate.

**Measurement of ROS production**

Cells (4 x 10^5^ cells/6-well plate) were grown with or without *O. octandra* extract (100 μg/ml) for 24 h and then 10 μM 2’,7’-dichlorofluorescin diacetate (H_2_DCFDA) (#D399, Molecular Probes Inc, Eugene, OR, USA) dissolved in DMSO was applied. After incubating at 37 °C for 20 min, the cells were detached and analyzed by flow cytometry (Beckman Coulter, Fullerton, CA, USA).

**Protein lysis and Western blot**

Cells (1 x 10^6^ cells/100-mm culture dish) were washed with cold PBS and lysed with Cell Lysis Buffer (#9803S, Cell Signaling Technology, Danvers, MA, USA) supplemented with PMSF (#78830. Cell Signaling Technology, Danvers, MA, USA). Protein (40-50 μg) were separated on 10% SDS-PAGE and transferred to PVDF membrane. The membranes were blocked in PBS with Tween 20 (PBST) containing 5% non-fat milk for 1 h at room temperature and incubated with appropriated primary antibodies at 4^o^C overnight. The primary antibodies against Fas (#8023s, 1:1000, Cell Signaling Technology, Danvers, MA, USA), Caspase-8 (#9746s, 1:1000, Cell Signaling Technology, Danvers, MA, USA), and Bid (#2002p, 1:1000, Cell Signaling Technology, Danvers, MA, USA) were used. After three washes with PBST, the membranes were incubated with secondary antibodies (anti-rabbit and anti-mouse-IgG, HRP-linked) which were described in original materials and methods, for 1 h. Protein bands were detected using chemiluminescence reagent (GenDEPOT, Barker, TX, USA).

**Statistical analysis**

All statistical analyses were analyzed by using the SPSS version 20 (SPSS Inc., Chicago, IL, USA). Mann-Whitney *U* test was used to determine the statistical significant difference. All of the variables were tested in three independent experiments, and each experiment was performed at least in triplicate. The results are shown as the mean ± standard deviation (SD). The value of **p* < 0.05 was considered statistically significant.
